# High-throughput screen for compounds that modulate neurite growth of human induced pluripotent stem cell-derived neurons

**DOI:** 10.1242/dmm.031906

**Published:** 2018-02-01

**Authors:** Sean P. Sherman, Anne G. Bang

**Affiliations:** Conrad Prebys Center for Chemical Genomics, Sanford Burnham Prebys Medical Discovery Institute La Jolla, CA 92037, USA

**Keywords:** Neurite growth, Human induced pluripotent stem cells, High-content screening

## Abstract

Development of technology platforms to perform compound screens of human induced pluripotent stem cell (hiPSC)-derived neurons with relatively high throughput is essential to realize their potential for drug discovery. Here, we demonstrate the feasibility of high-throughput screening of hiPSC-derived neurons using a high-content, image-based approach focused on neurite growth, a process that is fundamental to formation of neural networks and nerve regeneration. From a collection of 4421 bioactive small molecules, we identified 108 hit compounds, including 37 approved drugs, that target molecules or pathways known to regulate neurite growth, as well as those not previously associated with this process. These data provide evidence that many pathways and targets known to play roles in neurite growth have similar activities in hiPSC-derived neurons that can be identified in an unbiased phenotypic screen. The data also suggest that hiPSC-derived neurons provide a useful system to study the mechanisms of action and off-target activities of the approved drugs identified as hits, leading to a better understanding of their clinical efficacy and toxicity, especially in the context of specific human genetic backgrounds. Finally, the hit set we report constitutes a sublibrary of approved drugs and tool compounds that modulate neurites. This sublibrary will be invaluable for phenotypic analyses and interrogation of hiPSC-based disease models as probes for defining phenotypic differences and cellular vulnerabilities in patient versus control cells, as well as for investigations of the molecular mechanisms underlying human neurite growth in development and maintenance of neuronal networks, and nerve regeneration.

## Abstract (precis)

**Summary:** High-throughput, small molecule screening of hiPSC-derived neurons using a high-content, image-based approach focused on neurite growth identified hit compounds, including approved drugs, which target molecules or pathways known to regulate neurite growth.

## INTRODUCTION

New therapies for neurological indications have a high attrition rate, with only 8% ever making it to clinical trial (Miller, 2010). This statistic can be explained in part by a reliance on animal models, transformed cell lines and heterologous recombinant systems for drug discovery (Pankevich et al., 2014). Cell-based assays used in drug screening have historically depended on transformed lines because of their ease of culture, but these cells are generally aneuploid, and are limited in terms of how well they mirror human biology. However, use of more physiologically relevant primary cells is restricted by availability and inherent variability.

The advent of human induced pluripotent stem cell (hiPSC) technology has opened up the possibility of a scalable source of human cells to produce disease-relevant models for drug discovery. hiPSCs can be generated by reprogramming readily available cells including those from skin and blood, enabling derivation of lines representing numerous genotypes and disease phenotypes (Park et al., 2008; Takahashi et al., 2007; Yu et al., 2007). Once stably derived, they can be expanded indefinitely and differentiated to many different cell types that exhibit morphological and functional hallmarks of normal, albeit immature, human primary cells (Passier et al., 2016). To date, hiPSC have been used to model a growing list of neurological diseases, providing a proof of concept that their differentiated derivatives can recapitulate disease-associated pathologies; moreover, in some cases it has been shown that pathologies expressed by these cell-based disease models can be ameliorated by drugs known to be therapeutic for patients (Yu et al., 2013).

Development of technology platforms to perform compound screens of hiPSC-derived neural cells with relatively high throughput is essential to realize their potential for drug discovery. Technical challenges include scaled production of specific neural cell types in quality-controlled, cryopreserved lots and culturing in high-density assay formats suitable for robotic screening (Heilker et al., 2014). Here, we demonstrate the feasibility of high-throughput screening on hiPSC-derived neurons using a high-content, automated image-based approach focused on neurite growth, a process that is fundamental to the formation of neuronal networks and nerve regeneration (Al-Ali et al., 2017; Conde and Caceres, 2009).

We took advantage of the scalability of hiPSC to screen a collection of 4421 bioactive small molecules, which includes approved drugs, well-characterized tool compounds, natural products and human metabolites, on a commercial source of cortical-like hiPSC-derived neurons, iCell Neurons [Cellular Dynamics International (CDI)]. Based on an evaluation of multiple parameters including neurite, length, number and branching, as well as nucleus-based measures of overall cell health, we identified compounds that either promote or inhibit neurite growth, targeting molecules or pathways known to modulate neurites, as well as those not previously associated with neurite growth. These data define a subset of chemical probes for interrogating neurite growth, and provide validation of a platform for high-throughput screening of hiPSC-derived neurons.

## RESULTS

### Assay development and screening

**Fig. 1.**
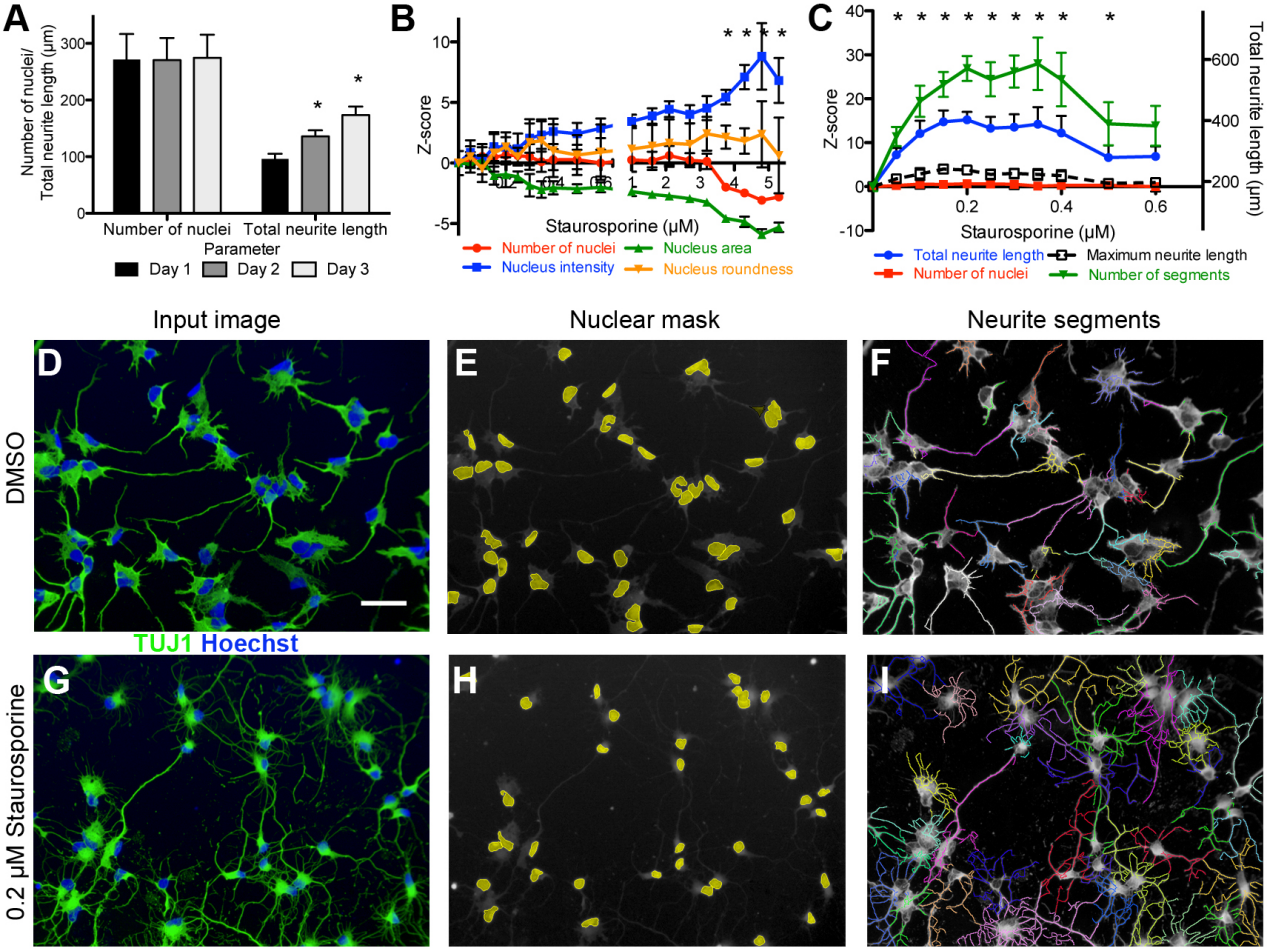
**High-content assay development on hiPSC-derived neurons**. (A-C) hiPSC-derived iCell Neurons are postmitotic and extend neurites rapidly after plating (A). Staurosporine treatment for 3 days was toxic at concentrations >3 µM (B), while a significant increase in total neurite outgrowth with no effect on cell health was observed at staurosporine concentrations between 0.05 and 0.5 µM (C). Data are mean±s.d., (A) *n*=32 wells (mean, 272 cells/well), (B,C) *n*=16 wells (mean, 288 cells/well), *P<0.001 by Student's *t*-test. Total neurite length corresponding to Z-scores is listed on the right axis for reference. Cell number and neurite morphology was measured using automated image analysis. (D-I) Input images (D,G) were separated into component color channels to identify nuclei (E,H; Hoechst, blue) and neurite segments (TUJ1, green). Neurite segments were then assigned to nuclei (F,I) in order to generate per cell measures of neurite morphology. Scale bar: 50 µm.

iCell Neurons, an hiPSC-derived, cortical-like cell population consisting primarily of γ-aminobutyric acid (GABA) interneurons, with a smaller contribution of layer V cortical pyramidal-like neurons (Berry et al., 2015; Dage et al., 2014; Meneghello et al., 2015), were used to develop a high-content assay for neurite outgrowth and inhibition. The broad-spectrum kinase inhibitor staurosporine, which promotes neurite outgrowth and branching at low concentrations (Hashimoto and Hagino, 1989), was used as a control compound to establish assay parameters in a 384-well format (Fig. 1). For image analysis, detection of nuclei was followed by neurite segment detection, and then neurite tree assignment to specific cells. An average of 240 total nuclei were analyzed per well. Values for 11 different parameters were calculated: four nuclear parameters (number of nuclei, nuclear area, roundness and fluorescence intensity) and seven neurite parameters [maximum and total neurite length, numbers of neurite extremities, segments, roots, nodes type 1 (points of intersection between two or more neurite segments) and nodes type 2 (number of neurite segments divided by number of roots)].

Assay conditions in which staurosporine is added 1 day after plating, followed by 3 days of incubation, resulted in Z-factors (Z’) for neurite parameters that ranged from 0.2 to 0.5, indicating that the assay was sufficiently robust for use in a high-throughput screen if run in duplicate to increase confidence in hit selection (Zhang et al., 1999). We found that laminin, an extracellular matrix protein known to support neurite growth (Baron-Van Evercooren et al., 1982), increased both the plating efficiency of cryopreserved cells and the number of neurites per cell in the assay when added to the plating medium at the time of thaw, without significantly affecting the detection window for outgrowth in response to staurosporine (data not shown). We also determined that iCell Neurons, both in terms of viability and outgrowth in response to staurosporine, are tolerant of dimethyl sulfoxide (DMSO) concentrations ≤0.5%, 10× higher than the vehicle concentration used for screening (data not shown).

We screened a collection of 5215 bioactive compounds, of which 4421 are unique, consisting of approved drugs, well-characterized tool compounds, natural products and human metabolites (Table 1). The approach of using small molecules with known targets allows us to identify specific biological pathways and molecular targets that modulate neurite growth and discover tool compounds for further exploration of the underlying biology.

**Table 1.**
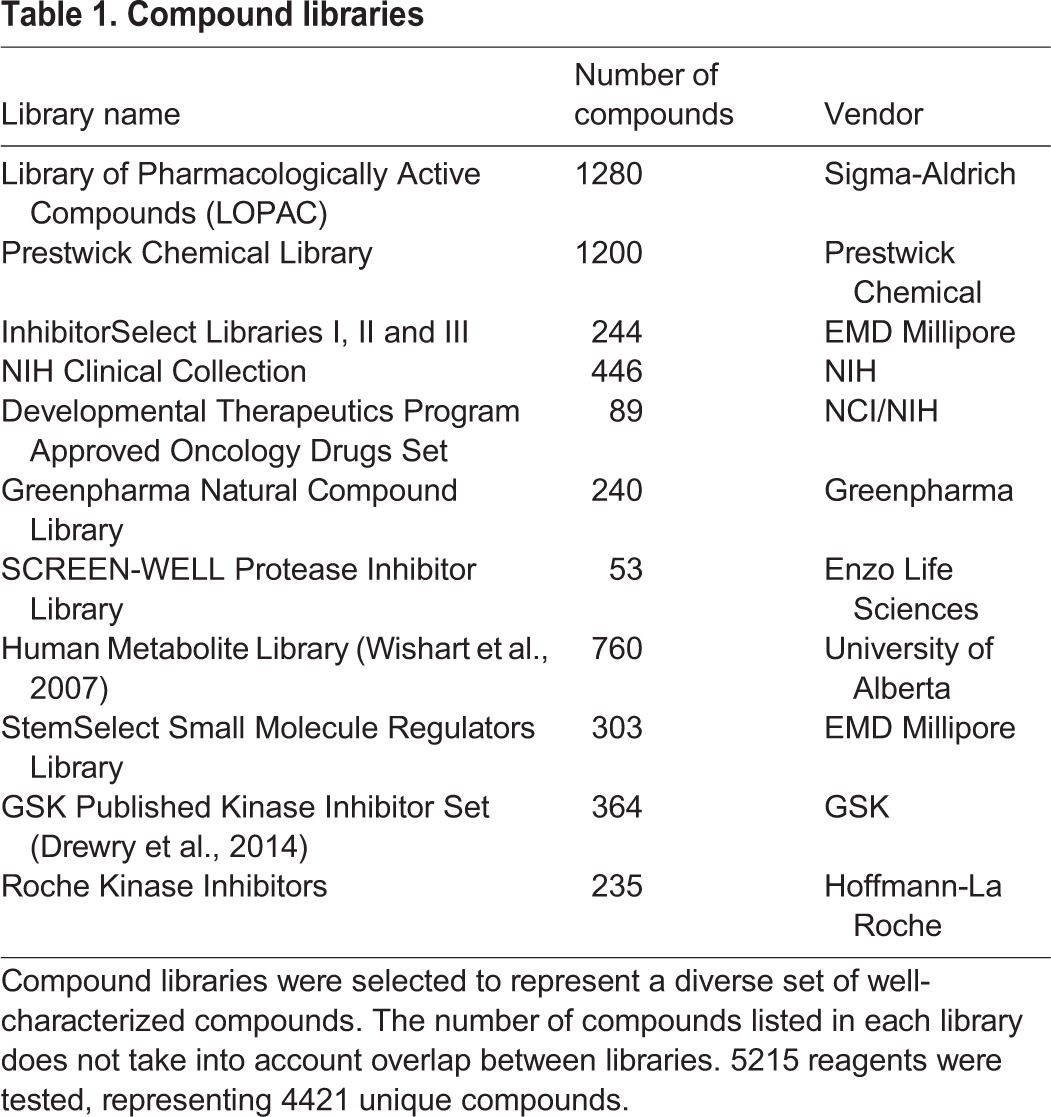
**Compound libraries**

**Fig. 2.**
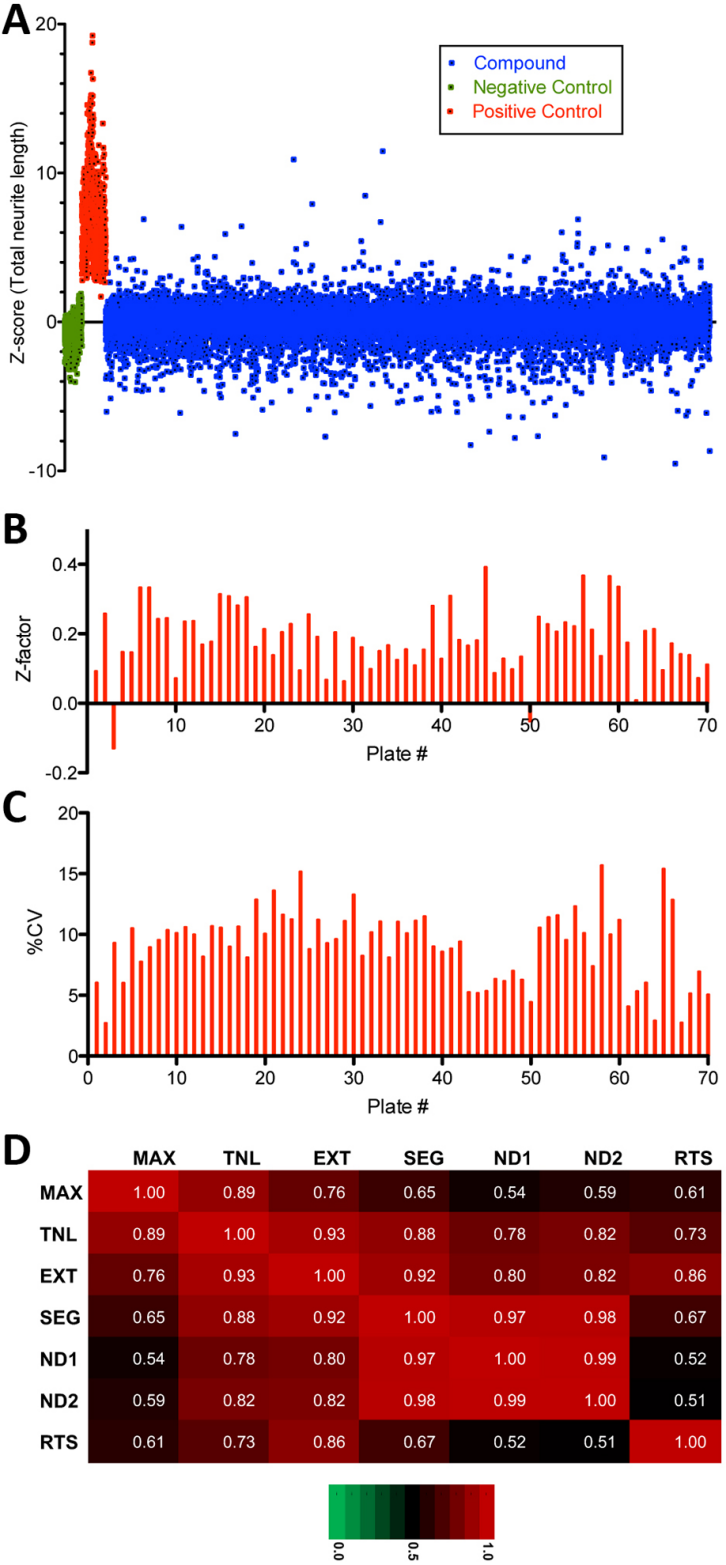
**Primary screen results overview.** (A) Scatter plot of primary screen data; 5215 compounds (blue) tested in duplicate at two concentrations (0.5 µM and 5.0 µM), for a total of 20,860 data points (blue, single replicates shown). Results were normalized to Z-scores based on DMSO-treated wells (green). Staurosporine at 0.2 µM (red) was used as a positive control. (B) Individual plate Z-factors for the primary screen range from −0.13 to 0.39 with an average of 0.19 (*n*=70), with only 2/70 plates exhibiting a Z-factor<0. (C) Coefficient of variation (CV) for screening plates ranges from 2.7% to 15.7%, with an average intraplate CV of 9.1% (*n*=70) and an interplate CV of 15.8%. (D) Coefficients of determination (r^2^) indicate that the seven measures of neurite morphology [MAX, maximum neurite length; TNL, total neurite length; EXT, number of extremities; SEG, number of neurite segments; ND1, number of nodes type 1 (intersection of segments); ND2, number of nodes type 2 (SEG divided by RTS); RTS, number of roots] are highly correlated, especially number of neurite segments (SEG) and both measures of number of nodes (ND1 and ND2) as they represent multiple readouts of neurite branching.

All compounds were screened in duplicate at two different concentrations, 0.5 µM and 5.0 µM, resulting in a total of ∼25,000 wells in a 384-well plate format, including controls (Fig. 2A). The screen performed acceptably well, according to criteria proposed by Zhang and colleagues (Zhang et al., 1999), with an average Z’ of 0.19 (Fig. 2B). Variability in the assay was limited, with an average intraplate coefficient of variation (CV) of 9.1% (Fig. 2C) and an interplate CV of 15.8%. For most hits, the seven neurite parameters monitored were highly correlated, suggesting general effects on neurite growth (Fig. 2D). In the first step of data analysis, we identified compounds that were overtly cytotoxic. A compound was considered cytotoxic at a Z-score≤−2 for number of nuclei or nuclear area, and ≥2 for nuclear fluorescence intensity or nuclear roundness, placing it outside of 95% of results in a normal distribution, in one or more nuclear parameters in duplicate wells. Using these criteria, we identified 172/4421 compounds (3.9%) that were toxic at both 5 µM and 0.5 µM. Another 111 (2.5%) were toxic at 5 µM, but not at 0.5 µM, and also had no effect on neurite measures at 0.5 µM. An additional 14 compounds were cytotoxic at 0.5 µM. In total, we eliminated 297 compounds (6.7%) from the list of potential hits owing to cytotoxic effects (Table S2).

We then used a similar approach to identify hits that either increased (Z score≥2.0) or decreased (Z-score≤2.0) any of seven neurite parameters in duplicate wells. In the primary screen, we identified 82 growth-promoting and 150 growth-inhibiting compounds, of which 50 and 58 confirmed in duplicate, respectively, in a repeat of the primary assay from cherry-picked compounds (Table 2; Tables S3 and S4). The lower confirmation rate for the growth-inhibiting compounds was due to cytotoxicity upon retest. In summary, screening resulted in an overall confirmed hit rate of 1% for compounds that increase neurite outgrowth and 1.3% for compounds that cause neurite retraction and/or inhibition of neurite growth.

**Table 2.**
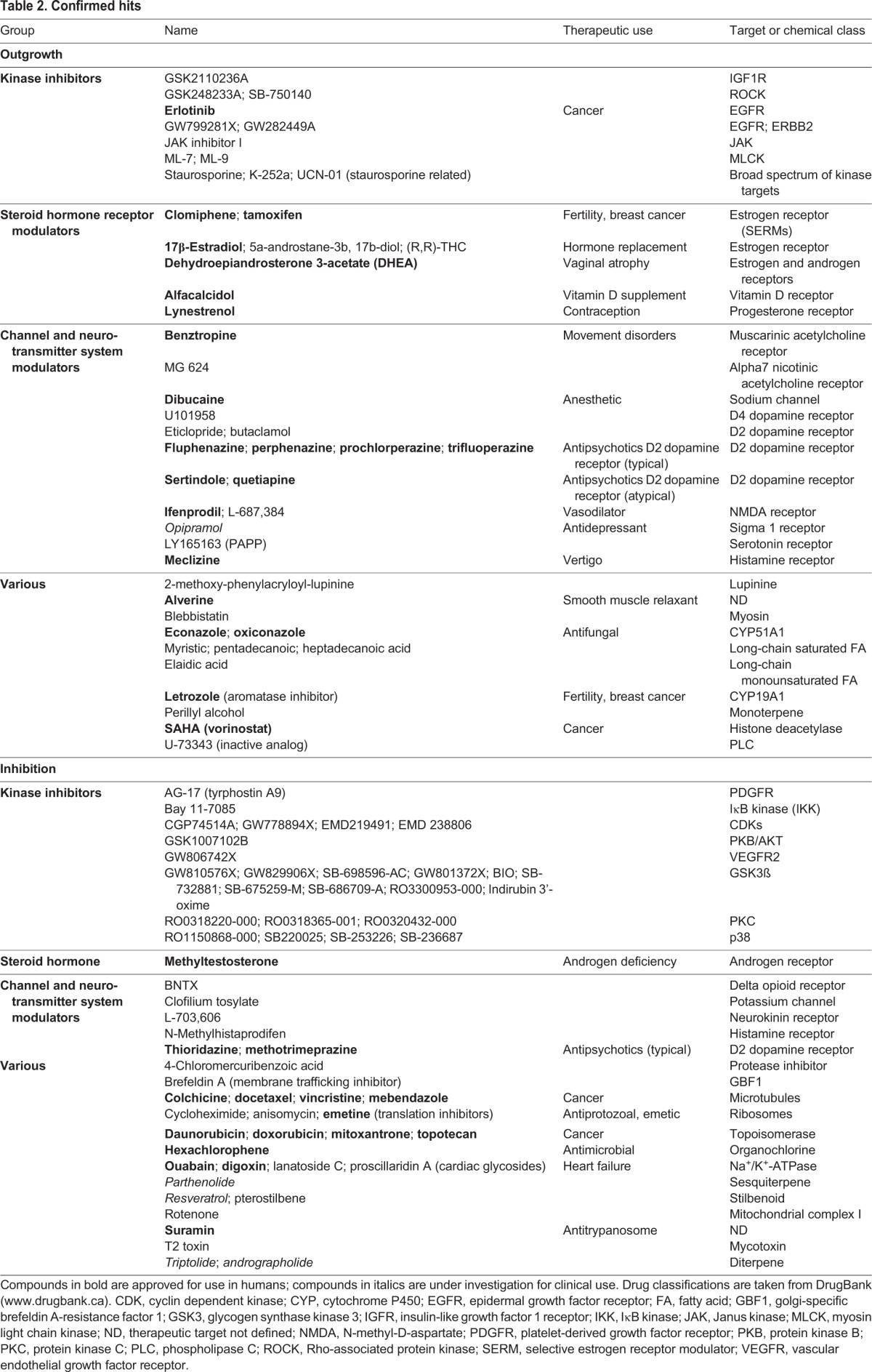
**Confirmed hits**

**Fig. 3.**
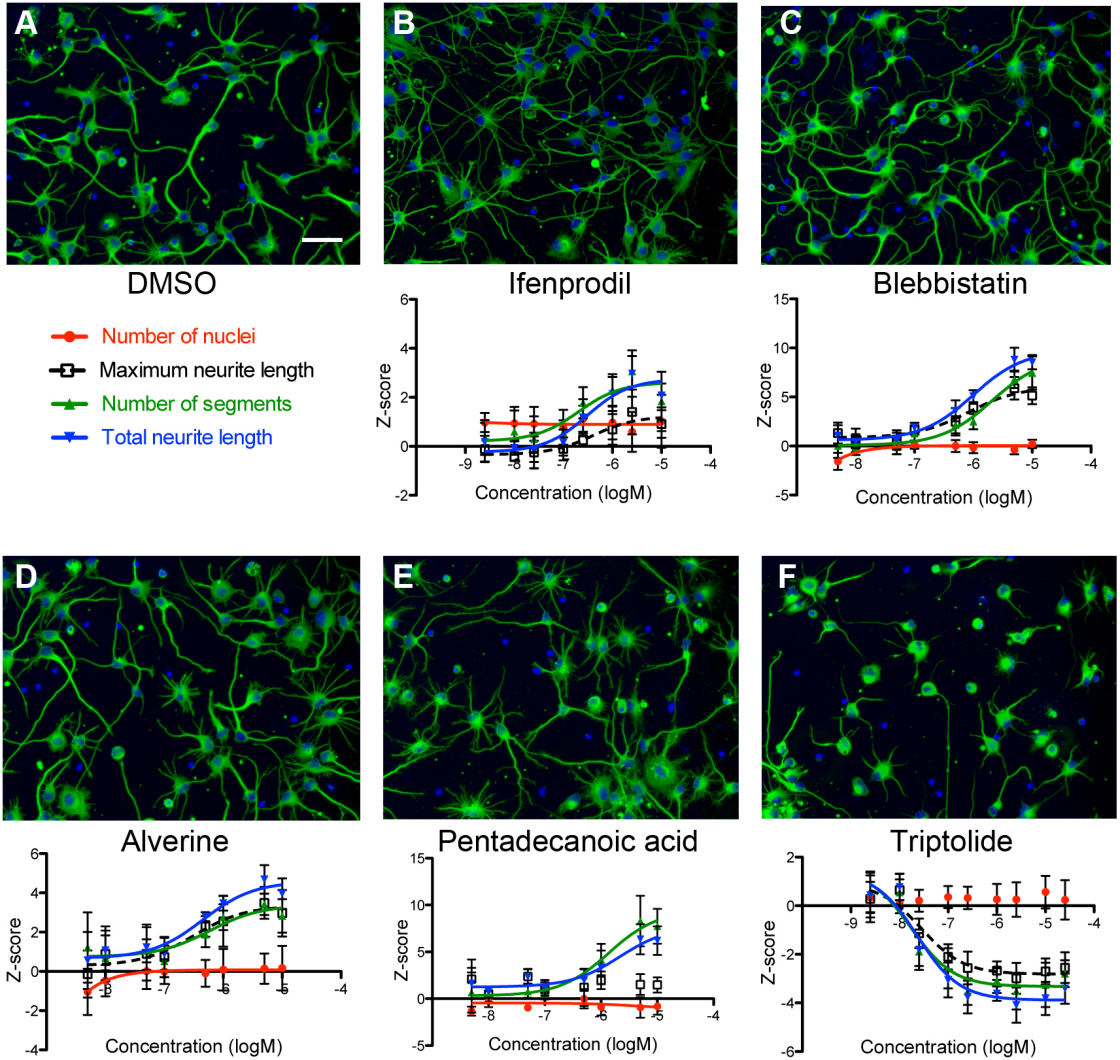
**Dose-response curves and images for confirmed screening hits.** (A-F) Treatment with the NMDA receptor antagonist ifenprodil (B, 5 µM), the myosin inhibitor blebbistatin (C, 5 µM), the smooth muscle relaxant alverine citrate (D, 5 µM), or the long chain fatty acid pentadecanoic acid (E, 5 µM) all increased measures of neurite growth compared to DMSO treated controls (A), whereas the plant-derived natural diterpene triptolide (F, 1 µM) reduces neurite outgrowth without affecting cell number. Scale bar: 50 µm. Data are mean±s.d., *n*=6 wells (mean, 244 cells/well).

Twenty-four representative compounds were selected for further confirmation, newly purchased as dry powders, solvated, and tested in a nine-point dose response at half-log concentration intervals. Dose-responsive effects were observed for multiple neurite measures; for a majority of these compounds the half-maximal inhibitory or effective concentrations, IC_50_ and EC_50_, respectively, were sub-micromolar, suggesting specificity (Fig. 3, Table 3; Fig. S1).

**Table 3.**
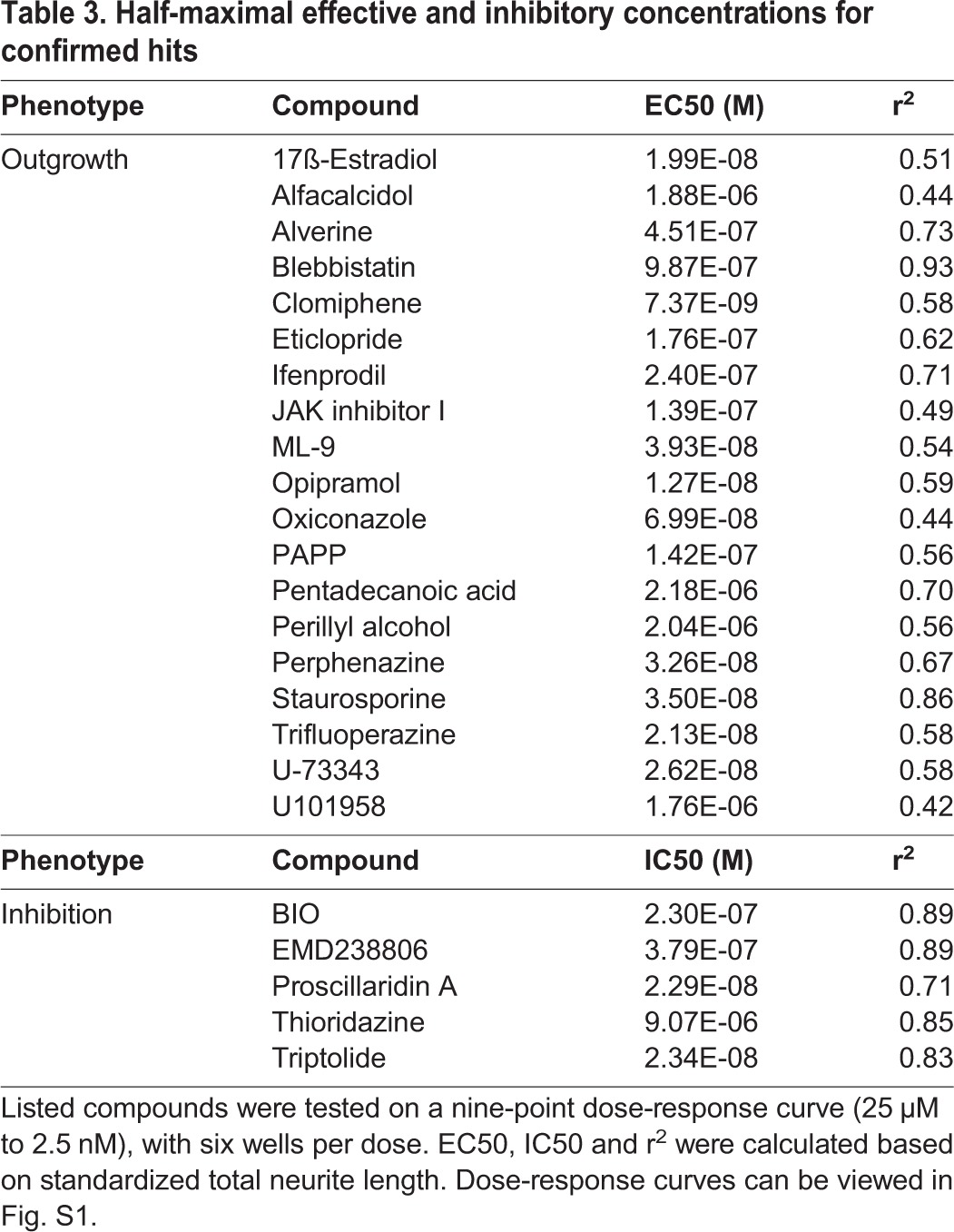
**Half-maximal effective and inhibitory concentrations for confirmed hits**

### Classification of screening hits

Confirmed hits were classified based on reported target, chemical class and/or therapeutic use, and then organized into four broad categories: kinase inhibitors, channel and neurotransmitter system modulators, steroid hormone receptor modulators and ‘various’ (Table 2). We noted that 21/50 (42%) of neurite growth-promoting and 16/58 (28%) of neurite-inhibitory hit compounds that we identified are approved drugs. We have cited reported targets for simplicity; however, it is important to take into consideration that compounds could have other activities and off-target effects, especially at higher concentrations used for screening (Arrowsmith et al., 2015). Many of the targeted pathways and some of the specific drugs and compounds that we identified have previously been implicated in the regulation of neurite growth in other systems based on transformed neural cell lines and/or rodent primary neurons. We also identified a number of compounds that would not have been predicted to impact neurite growth from published reports. Below we discuss hit classes in the context of their reported targets and known roles in neurite growth, highlighting kinase inhibitors, modulators of neurotransmitter systems and modulators of steroid hormone receptors.

### Kinase inhibitors

As expected, our positive control compound, staurosporine, and its closely related analogs UCN-01 and K-252a, were identified as hits that promote neurite outgrowth. These compounds inhibit a wide spectrum of kinases, including protein kinase C (PKC; PRKC proteins), but the mechanism underlying their effects on neurite growth is unknown (Thompson and Levin, 2010). Our results are consistent with reports that staurosporine's effects on neurites occur via a PKC-independent mechanism (Rasouly et al., 1992), as we identified three PKC inhibitors that inhibited neurites, RO0318220-000, RO0318365-001 and RO0320432-000, which, moreover, were originally developed to improve specificity of staurosporine towards PKC (Bit et al., 1993; Davis et al., 1994).

Another expected group of kinase inhibitors we identified included modulators of myosin II, which plays a central role in neurite outgrowth and retraction (Newell-Litwa et al., 2015; Schmidt et al., 2002). Myosin II is regulated through phosphorylation of myosin regulatory light chain (MLC; MYL proteins), which leads to increased contractility and growth cone collapse. Hits in this group included ML-7 and ML-9, inhibitors of myosin light chain kinase (MLCK; MYLK), and GSK248233A and SB-750140, inhibitors of Rho-associated kinase (ROCK) proteins, which have a dual role, acting to phosphorylate and activate MLC, and to phosphorylate and inhibit myosin light chain phosphatase (MLCP). We also identified blebbistatin, an inhibitor of myosin II ATPase activity. In support of these findings, blebbistatin has been previously shown to promote neurite outgrowth on avian primary neurons (Rösner et al., 2007) and hiPSC-derived neurons (Boissart et al., 2013), and neurite growth-promoting activity by ML-7 and SB-750140 has been demonstrated in rodent primary neurons (Al-Ali et al., 2015, 2013).

**Fig. 4.**
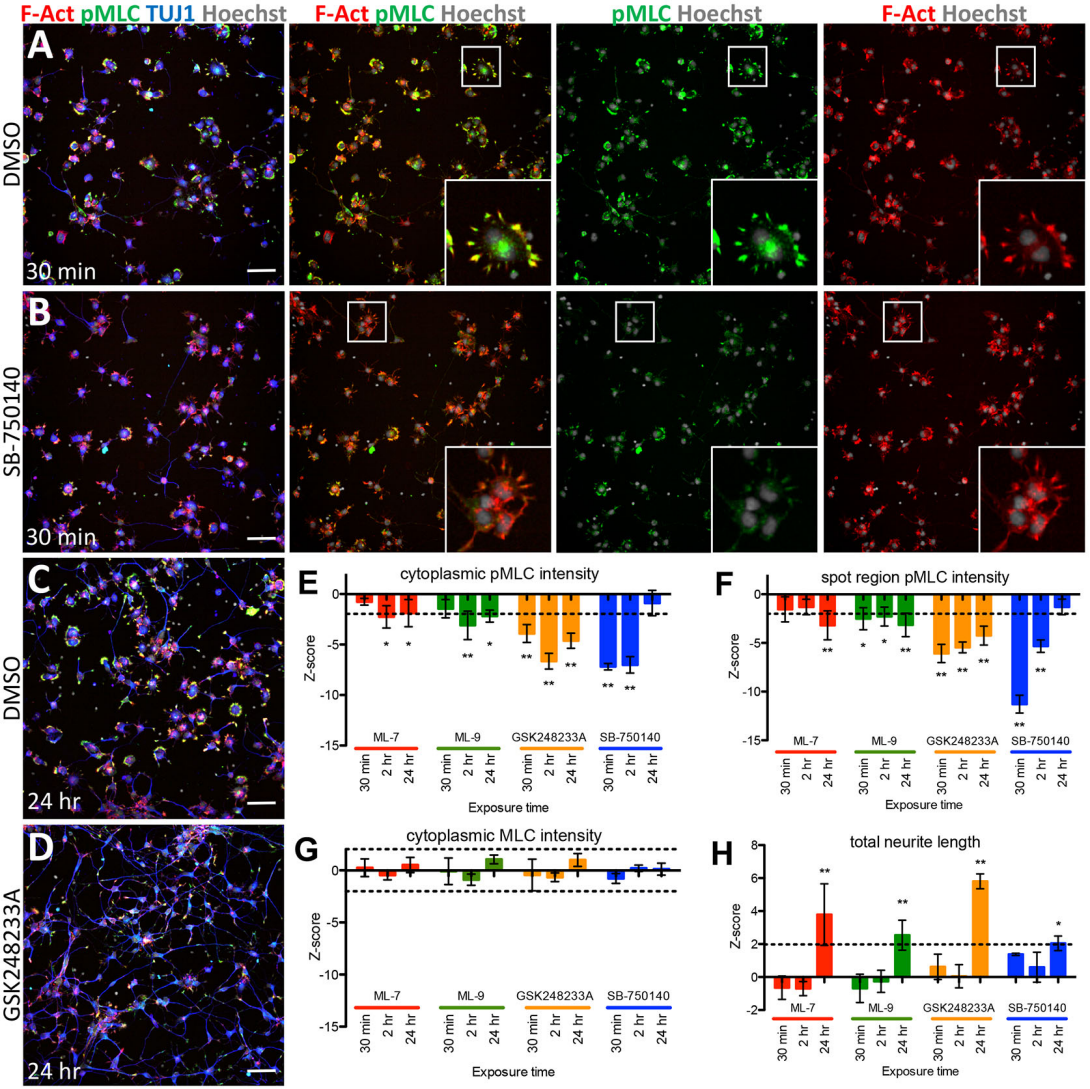
**Inhibitors of ROCK and MLCK affect neurite growth via regulation of MLC phosphorylation.** (A-D) Representative images of compound-treated cells. Treatment with the ROCK inhibitor SB-750140 (B) at 5 µM resulted in a reduction in pMLC (green) compared to DMSO-treated cells after 30 min of compound exposure (A,B). The reduction in pMLC was observed in particular at points of cell spreading and neurite initiation, highlighted by phalloidin-stained F-actin (red, insets). Treatment with the ROCK inhibitor GSK248233A (D) at 5 µM resulted in an increase in total neurite length [TUJ1-stained neurites (blue)] and decrease in pMLC (green) compared to DMSO-treated cells at 24 h of compound exposure (C,D). Scale bars: 50 µm. (E-H) Quantification of pMLC and MLC fluorescent intensities, and total neurite lengths. Significant reduction in pMLC was observed after treatment with two ROCK inhibitors at 5 µM (ML-7 and ML-9) and two MLCK inhibitors at 5 µM (SB-750140 and GSK248233A). This reduction was observed in both cytoplasmic pMLC levels (E) and in pMLC at concentrated regions of F-actin localization (F), while overall levels of total MLC were unchanged (G). Effects on pMLC were observed within 24 h of compound treatment, at which point significant neurite outgrowth was also observed (H). Data are mean±s.d., *n*=4 wells per condition (mean, 1211 cells per well), *P<0.05, **P<0.001, by Student's *t*-test. Dashed lines indicate the threshold of Z=±2.

Our classification of GSK248233A and SB-750140 as likely ROCK inhibitors is based on a study characterizing the GlaxoSmithKline Published Kinase Inhibitor Set (GSK PKIS) library against ∼50% of the human kinome (Elkins et al., 2016). To confirm activities of GSK248233A and SB-750140, as well as ML-7 and ML-9, against their reported targets in our high-content assay, we monitored phosphorylated MLC (pMLC) expression at several time points after compound treatment, in the cytoplasm, and, in particular, in spots that colocalized with areas of phalloidin-stained F-actin corresponding to filopodia at points of cell spreading, neurite initiation and growth cones (Fig. 4A-D), as described for rodent and chicken primary neurons (Kollins et al., 2009; Kubo et al., 2008; Yu et al., 2012). As expected for inhibitors of MLCK, we observed that ML-7 and ML-9 decreased pMLC levels, an effect that peaked at 24 h after compound exposure (Fig. 4E,F), similar to a previous report on the effect of ML-7 on chicken embryonic primary neurons (Kollins et al., 2009). Consistent with inhibition of ROCK-mediated phosphorylation of MLC and/or MLCP, GSK248233A and SB-750140 also decreased pMLC levels, with strong effects observed at 30 min and 2 h, which diminished by 24 h (Fig. 4A-F). Total MLC levels did not change in response to compound treatment across all time points (Fig. 4G). Corresponding increases in total neurite length were observed by 24 h for all four compounds tested (Fig. 4C,D,H). Together, these data are consistent with ML-7, ML-9, GSK248233A and SB-750140 affecting neurite growth through inhibition of their reported targets. Moreover, they underscore the value of using automated image-based approaches to study regulatory pathways at a subcellular level, and demonstrate the utility of our high-content assay for target and pathway validation.

The largest kinase inhibitor class we identified inhibited neurites and consisted of 10 glycogen synthase kinase 3 beta (GSK3β) antagonists (Table 2). In the regulation of neurite growth and axiogenesis, both positive and negative roles have been attributed to GSK3β and the closely related enzyme GSK3α in rodent models (Kim et al., 2011). Our observation that inhibition of GSK3β inhibits neurites is consistent with multiple reports on neuronal cell lines and rodent primary neurons, including one proposing that GSK3β inhibition mimics the inhibitory effects of myelin on neurite outgrowth (Alabed et al., 2010). Other reports suggest that neurite response to GSK3 inhibition depends on developmental stage and GSK3 activity level. In one study, strong knockdown of GSK3α and GSK3β activity reduced axon growth, while moderate reduction resulted in axon branching (Kim et al., 2006), whereas others reported that axon length is reduced when GSK3 is inhibited during initiation of axon growth, but branching is increased when GSK3 is inhibited after an axon is already specified (Castaño et al., 2010; Garrido et al., 2007).

**Fig. 5.**
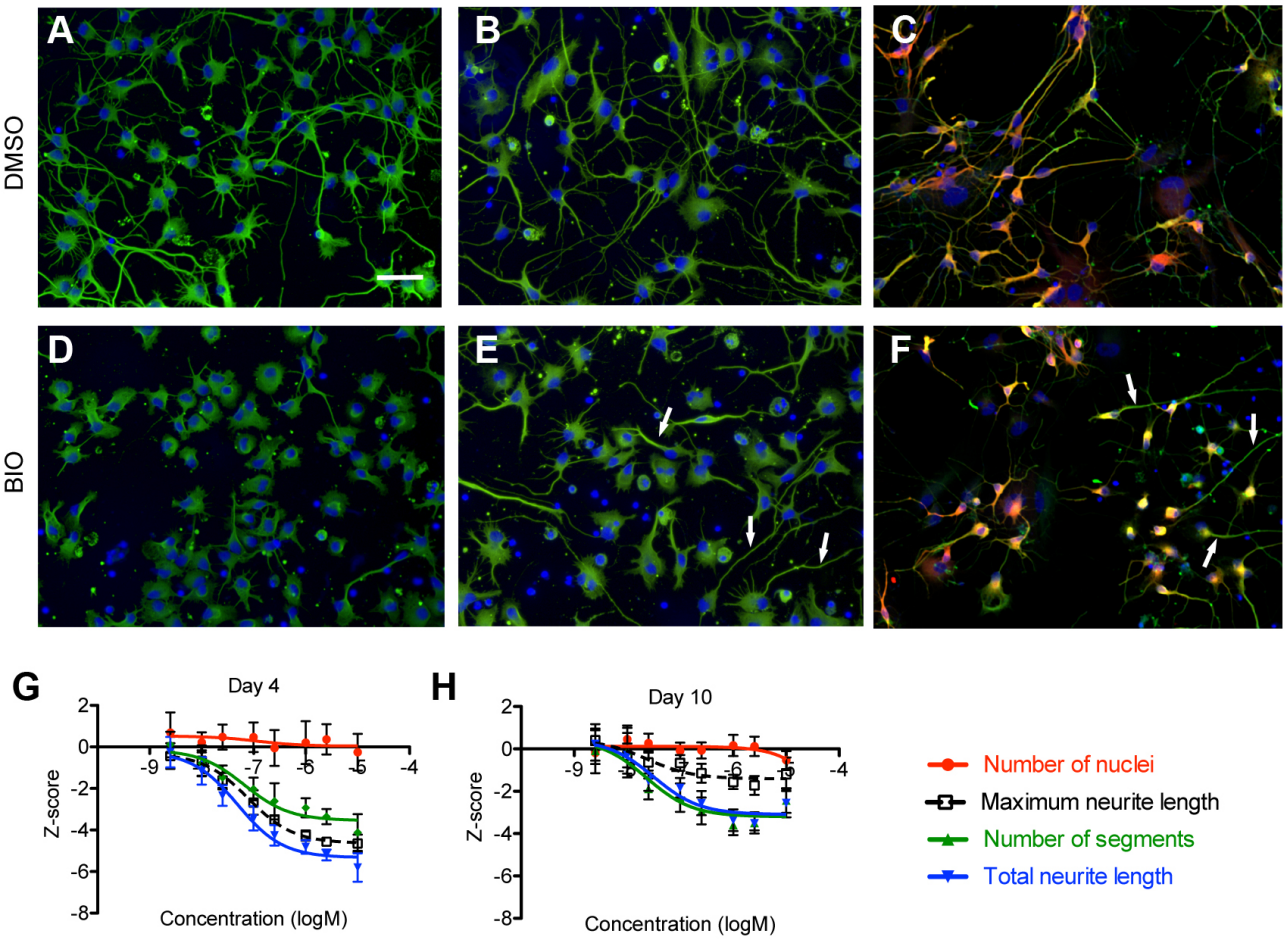
**BIO promotes neurite retraction.** (A-H) In the primary screen, BIO was identified as a compound that reduced neurite outgrowth after adding compound to neurons 1 day postplating and incubating further for 3 days (A,D). To test the effects of BIO on more developed neurites, neurons were cultured for 7 days after plating before treating with BIO for 3 days (10 days total in culture) (B,E). In both cases, neurons treated with 2.5 µM BIO had reduced neurite growth compared to vehicle-treated wells (DMSO). Treatment with BIO resulted in a dose-dependent decrease in neurite length without cytotoxic effects at either DIV4 (G) or DIV10 (H). Data are mean±s.d., *n*=6 wells (mean, 248 cells/well). In long-term culture (DIV14), hiPSC-derived neurons generated dendrites that are co-positive for MAP2 (red) and TUJ1 (green), as well as TUJ1+MAP2- axons (C,F). Treatment with 2.5 µM BIO results in retraction of double-positive dendrites, but sparing of MAP2-negative/TUJ1-positive axons (arrows). Scale bar: 50 µm.

In our dose-response experiments, addition of the GSK3β inhibitor, BIO, at 1 day in vitro (DIV1), and assay at DIV4 resulted in dose-dependent decreases of all neurite measures, without increased branching at lower concentrations (Fig. 5A,D,G). However, adding BIO at a later time point, DIV7, after considerable neurite growth has already occurred, and assaying at DIV10 resulted in a clear dose-dependent decrease in all neurite measures, except for maximum neurite length, for which the reduction was blunted (Fig. 5B,E,H). Analyses of these cultures for expression of MAP2, a dendrite-specific marker, and β-tubulin, which marks all neurites, suggest that the longest neurite is an axon (Fig. 5C,F). Taken together, these results are consistent with the hypothesis that inhibition of GSK3β activity at early time points (DIV1) reduces neurite growth, but GSK3β inhibition at a later time point (DIV7) promotes dendrite retraction and possibly reduces growth, but spares axons which have passed a critical window of developmental specification. Given the prominence of GSK3 as a target for the development of drugs to treat neuropsychiatric and neurodegenerative disease (Mulligan and Cheyette, 2017; Seira and Del Rio, 2014; Singh, 2013), our analyses suggest that hiPSC-derived neurons could be useful models to test these drugs, especially in the context of patient-specific genetic backgrounds. These results also underscore the utility of hiPSC for modeling human neurons at different states of maturity, towards understanding differential effects of drugs on newborn neurons generated during adult neurogenesis, for instance in the hippocampus, versus mature neurons (Valvezan and Klein, 2012).

We also identified a number of other kinase inhibitors that target intracellular signaling pathways. These included only one compound that promoted neurite outgrowth, a Janus kinase (JAK) protein inhibitor, consistent with reports that JAK-STAT mediated signaling is a negative regulator of this process (Gupta et al., 2011). The remaining hits we identified in this group were inhibitory to neurites: four cyclin dependent kinase (CDK) protein inhibitors, as well as antagonists of p38 mitogen-activated protein kinase (MAPK), IκB kinase (IKK) and AKT proteins, kinases previously reported to regulate neurite growth in other systems (Gutierrez et al., 2005; Iwasaki et al., 1999; Morooka and Nishida, 1998; Nikolic et al., 1996; Read and Gorman, 2009).

Finally, we identified multiple receptor tyrosine kinase (RTK) inhibitors that either promoted [GSK2110236A, and three epidermal growth factor receptor (EGFR) inhibitors], or inhibited (GW806742X and tyrophostin-A9) neurite outgrowth. The GSK PKIS library compounds GSK2110236A and GW806742X were originally reported as insulin-like growth factor 1 receptor (IGFR) and vascular endothelial growth factor receptor (VEGFR) inhibitors, respectively; however, upon further characterization, these compounds were shown to exhibit extensive off-target activity, including a range of other RTKs, such that a likely target cannot be predicted (Elkins et al., 2016). The three EGFR inhibitors we identified that promoted neurite outgrowth included erlotinib, and two GSK PKIS library compounds, GW799281X and GW282449A, which are reported to also target the closely related RTK, ERBB2 (Elkins et al., 2016). Pharmacological inhibition of the EGFR, including by erlotinib, has been shown to promote neurite outgrowth *in vitro* on rodent primary neurons, but only in the presence of inhibitory cues that block axon regeneration after injury, such as chondroitin sulfate proteoglycans (CSPGs) (Koprivica et al., 2005; Leinster et al., 2013). Similar observations after nerve injury *in vivo* have motivated efforts to target inhibition of the EGFR to promote axonal regeneration (Koprivica et al., 2005; Vigneswara et al., 2012). Interestingly, recent studies have demonstrated that herceptin-mediated blockade of ERBB2 also enhances nerve regeneration after injury *in vivo* by inhibiting transactivation of the EGFR (Hendry et al., 2016). In summary, in contrast to reported studies on rodent neurons *in vitro*, we identified multiple small molecule inhibitors of the EGFR that promoted neurite outgrowth in the absence of added inhibitory cues, a difference that could be explained by species, neuronal subtype or maturity, or the possibility that CSPGs or other inhibitory matrix components might be expressed by hiPSC-derived neurons.

### Steroid hormone receptor modulators

Another major class of neurite growth-promoting compounds that we identified was steroid hormone receptor modulators. Several of these were estrogenic, including 17β-estradiol and dehydroepiandrosterone (DHEA), neurosteroids that are known to promote neurite growth (Arevalo et al., 2012; Maninger et al., 2009). We also identified tamoxifen and clomiphene, selective estrogen receptor modulators (SERMs), which as therapeutics primarily act as ER antagonists; however, they can also exhibit partial agonist activity in a concentration- and cell type-specific manner (Haskell, 2003), which could explain their neurite growth-promoting activity in our assay. We noted another compound that we identified as neurite growth promoting that might act as an estrogen receptor agonist: U-73343, an inactive analog of the protein lipase C (PLC proteins) inhibitor U-72122, described in a previous study as possessing strong estrogenic activity (Cenni and Picard, 1999).

In addition, we identified two other steroid hormones that promoted neurite growth: vitamin D3, similar to a previous report for rodent primary neurons (Brown et al., 2003), and a progestogen, lynestrenol. Progestogens are known to promote neurite growth (Wessel et al., 2014). However, lynestrenol is a synthetic pro-drug that needs to be metabolized to an active form (Korhonen et al., 2008), suggesting the presence of an appropriate cytochrome P450 (CYP) in hiPSC-derived neurons. CYP2C19 would be a candidate, as it both metabolizes lynestrenol and is expressed in human fetal brain (Korhonen et al., 2008; Persson et al., 2014). Finally, we identified two androgenic compounds: one that inhibited neurites, methyltestosterone, and one that increased neurite growth, the azole letrozole. Methyltestosterone is a synthetic anabolic steroid, which has been shown previously, at high concentrations, to inhibit neurites on PC12 cells (Basile et al., 2013). Letrozole is an inhibitor of the aromatase CYP19A1, which converts androgen to estrogen. Our identification of letrozole is consistent with reported neurite growth-promoting activities associated with androgens (Estrada et al., 2006; Marron et al., 2005; Reddy et al., 2015), and implies that CYP19A1, which is expressed in fetal brain (Montelli et al., 2012), is expressed in hiPSC-derived neurons. Alternatively, letrozole could have off-target effects in our assay. Indeed another neurite growth-promoting azole we identified, econazole, which inhibits fungal CYPs, has been shown to promote neurite growth in rodent neural cell lines and primary neurons through an off-target effect on the calcium channel transient receptor potential cation channel 2 (TRPM2) (Jang et al., 2014). Taken together, these results suggest that hiPSC-derived neurons could be useful models to investigate regulation of human brain development, homeostasis and disease by steroid hormones.

### Neurotransmitter system modulators

A significant number of hit compounds that promoted neurite growth (14/50) targeted neurotransmitter receptors. The majority of these hits target dopamine receptors: 8/14 are antagonists of D2 dopamine receptors (D2R), and one, U101958, has been shown to act as an agonist of the D4 dopamine receptor (Schlachter et al., 1997). The D2R hits included the tool compounds, eticlopride and butaclamol, as well as the atypical antipsychotics, sertindole and quetiapine, which have been shown to promote neurite outgrowth in PC12 cells (Lu and Dwyer, 2005). We also identified the typical antipsychotics and D2R antagonists, fluphenazine, perphenazine, prochlorperazine and trifluoperazine. These drugs were previously described as neurite growth promoting in rat hippocampal primary neurons, but only when plated on nonpermissive substrates such as CSPGs (Johnstone et al., 2012). By contrast, we observed growth-promoting activity on the permissive substrate laminin, a difference that could be attributed to many factors including species, neuronal subtype and maturity, and the possibility, as discussed above, that CSPGs or other inhibitory matrix components are expressed by hiPSC-derived neurons. In addition to the eight D2R antagonists which promoted neurite outgrowth, we also identified two D2R antagonists, thioridazine and methotrimeprazine, which inhibited neurites in our assay, results consistent with a previous study on rodent primary neurons (Johnstone et al., 2012), as well as with reports that D2 receptor stimulation can enhance neurite growth (Todd, 1992).

We did not observe a consistent relationship between dopamine receptor modulators we identified in our screen and neurite growth: eight of the D2 receptor antagonists promoted neurite growth and two were inhibitory. It is very likely that at least some of our findings reflect off-target activities or combinations of targets. For instance, the D2-antagonist antipsychotics are known to also modulate, with lower affinity, other neurotransmitter receptors (Miyamoto et al., 2012). As well, it has been proposed that D2R antagonists of the phenothiazine class, such as the typical antipsychotics we identified, promote neurite growth through an off-target effect on calmodulin (Johnstone et al., 2012).

We also identified neurite growth-promoting compounds that target cholinergic (both muscarinic and nicotinic), histamine and serotonin receptors, neurotransmitter systems previously implicated in neurite growth (Dwyer et al., 2008; King and Kabbani, 2016; Lipton et al., 1988; Munis et al., 1998; Shideler and Yan, 2010; Wirth et al., 2017). We note that our identification of the muscarinic receptor antagonist benztropine is opposite to expectations based on reports demonstrating positive roles for muscarinic receptor activation in neurite growth (Shideler and Yan, 2010; VanDeMark et al., 2009). Further analyses are required to identify the targets of these drugs in our system and determine whether the effects of the hit compounds reflect off-target activities, and the extent to which they are species or neuronal cell subtype- or maturation stage-specific.

A potential common mechanism of action among a number of the neurotransmitter receptor and steroid hits that were identified as promoting neurite outgrowth in our screen, might be through modulation of the sigma-1 receptor (σ_1_R). The σ_1_R is a novel endoplasmic reticulum (ER) chaperone, which regulates a diversity of cellular functions, including neuritogenesis (Rousseaux and Greene, 2015). Binding to σ_1_R and activation by a range of psychotropic drugs, and its ability to modulate multiple neurotransmitter systems has led to an intense interest in its role in neurological disease and as a drug target in neuropharmacology (Hayashi, 2015). The mechanism by which the σ_1_R promotes neurite outgrowth is unclear, but evidence suggests that its activation stabilizes inositol triphosphate (IP3), causing increased calcium influx, which leads to increased tricarboxylic acid (TCA) cycle and energy production, and thus promotes neurite outgrowth and neuroprotective effects (Ishikawa and Hashimoto, 2010). The NMDA receptor antagonist, ifenprodil, which we identified in our screen, has been previously shown to promote neurite outgrowth by a mechanism involving σ_1_R rather than the NMDA receptor (Ishima and Hashimoto, 2012). In addition to ifenprodil, a number of other hits we identified that promote neurite outgrowth are ligands of the sigma-1 receptor including the NMDA receptor antagonist L-687384 (McLarnon et al., 1994), the psychotropic compound opipramol (Ferris et al., 1991), the dopamine receptor modulators quetiapine (Kotagale et al., 2013) and U101958 (Helmeste et al., 1999), the SERMs tamoxifen and clomophene (Moebius et al., 1997), and the steroid hormone DHEA (Su et al., 1988). Whether these compounds promote neurite outgrowth through the σ_1_R on hiPSC-derived neurons will be tested in future studies.

### Drugs known to cause neuropathies or other neurotoxic effects

Several classes of approved drugs that we identified as inhibiting neurites are known to cause peripheral neuropathies or other neurotoxic side effects. We distinguish these hits from those that are overtly toxic in that they inhibited neurites at concentrations at which we did not detect effects on cell number or nuclear parameters as measures of cell health. One such class was cardiac glycosides, inhibitors of the Na^+^/K^+^-ATPase, which have been shown to inhibit neurite outgrowth in cell culture models and cause neuropathies in animal models, phenotypes thought to result from buildup of intracellular sodium (Penniyainen et al., 2009; Persson et al., 2013). Another drug we identified, the antiseptic hexachlorophene, was restricted by the US Food and Drug Administration to prescription use only, after it was associated with brain damage (Evangelista de Duffard and Duffard, 1996). Consistent with our results, hexachlorophene was also shown in a previous, focused screen of 80 known and potential toxins to cause neurite retraction in hiPSC-derived neurons (Ryan et al., 2016).

We also identified several anticancer drugs with neuropathic side effects. These included DNA intercalators such as doxorubicin, a widely used chemotherapeutic, which causes eviction of histones and inhibits topoisomerase II, leading to dysregulation of DNA damage response and transcription (Nitiss, 2009). Crucial roles for topoisomerases in neurite growth have been demonstrated in knockout mice, and in rodent primary neurons treated with doxorubicin and other topoisomerase inhibitors (Manchon et al., 2016; Meiners et al., 2007; Nitiss, 2009; Tsutsui et al., 2001). However, in clinical studies, although doxorubicin has been associated with cognitive dysfunction, it is unclear whether sufficient levels pass the blood-brain barrier to act directly on neurons or whether these effects are indirect (Manchon et al., 2016; Rousselle et al., 2001; Tacar et al., 2013). Another anticancer drug we identified as an inhibitor of neurites is suramin, a P2 receptor antagonist and antitrypanosome agent (McGeary et al., 2008). Suramin is associated with peripheral neuropathies when used as a chemotherapeutic for metastatic prostate cancer, and has been shown to block neurite outgrowth in neural cell lines and cause axonal degeneration in animal models (Cui et al., 2011; Lakshmi and Joshi, 2006; Russell et al., 2001; Sioka and Kyritsis, 2009). Finally, we identified chemotherapeutic microtubule inhibitors, such as vincristine, which are associated clinically with peripheral neuropathies. Vincristine has been shown previously to inhibit neurites in cultured rodent primary neurons and neural cell lines (Krug et al., 2013; Radio and Mundy, 2008; Sioka and Kyritsis, 2009), as well as hiPSC-derived neurons (Wheeler et al., 2015), in a study that proposed that patient-specific iPSCs will be useful to better understand genetic vulnerabilities to vincristine-associated neuropathies.

Whether neurite inhibition caused by the compounds we identified in our screen is tolerated, but, for instance, leads to synaptic or network impairment, or alternatively, is a harbinger of irreversible cell damage and eventual cell death, has yet to be determined. Analyses of neurite recovery after compound removal, as well as the effects of longer term compound exposure and repeat dosing, could help address these questions, and will be interesting to compare with *in vivo* studies, for development of hiPSC cell-based assays for predictive toxicology.

### Novel findings

Although many of the hits we identified, or the pathways they target, have been previously implicated in neurite growth, some hits would not have been predicted. These include three natural products: 2-methoxy-phenylacryloyl-lupinine, which promoted neurite outgrowth, and two Chinese herbal medicines, the diterpines, andrographolide and triptolide, which inhibited neurites. Andrographolide has been shown to inhibit GSK3β and NF-κB proteins (Varela-Nallar et al., 2015; Zhang et al., 2014), and triptolide has been shown to activate ROCK and promote MLC and MYPT protein phosphorylation, findings that could explain their neurite inhibitory activities in our assay (Gutierrez et al., 2005; Liu et al., 2013). Another hit not previously implicated in neuritogenesis was the smooth muscle relaxant alverine citrate, which promoted neurite outgrowth in our screen. Alverine citrates's mechanism of action is not well understood, but it has been proposed to antagonize 5HT1A receptors, and also regulate calcium influx and ROCK activity, potential routes for promoting neurite outgrowth (Coelho et al., 2001; Gupta et al., 2014; Hayase et al., 2007; Nikolic, 2002; Rojas et al., 2014).

We also identified three long-chain saturated fatty acids (FAs) as novel neurite growth-promoting hits. These FAs are dietary (exogenous) metabolites present in the human metabolite library we screened. Endogenous and dietary FAs play important roles in brain health and disease (Lei et al., 2016), and although previous reports found that long-chain polyunsaturated FAs, and medium-chain saturated FAs promoted neurite growth in rodent neural cell lines (Darios and Davletov, 2006; Kamata et al., 2007; Marszalek et al., 2004), to our knowledge, our identification of long-chain saturated FAs as neurite growth promoting is novel. Finally, we identified a number of compounds for which activity was opposite to that expected from previous reports. These include the sodium-channel blocker dibucaine identified as neurite outgrowth promoting in our screen, but shown previously to inhibit neurites (Kasaba et al., 2003), and the polyphenol resveratrol, previously reported to promote neurite outgrowth in N2a and PC12 cells (Dasgupta and Milbrandt, 2007; Sugino et al., 2010), but identified in our screen as inhibitory to neurites, differences that could be explained by species, neuronal subtype, or maturity of the neurons used in these studies.

## DISCUSSION

Development of patient-specific hiPSC-based models to study the cellular and molecular bases of neurological disease offers an opportunity to identify novel drugs and improved treatments. We have demonstrated the feasibility of high-throughput phenotypic screening of hiPSC-derived neurons, an important step towards realizing their potential in drug discovery. Indeed, phenotypic screening for small molecules that modulate a cellular phenotype, interrogating all components and pathways of the cell and not just a single target, is an approach that has been remarkably effective at producing drug candidates (Swinney and Anthony, 2011).

The high-throughput assay platform we established can be used to interrogate fundamental aspects of neuronal morphology, and also provides a basis for further development of more complex phenotypic readouts for target/pathway validation and compound screens based on patient hiPSC-derived neural cell types. Such high-throughput, miniaturized assays will be advantageous not only for drug screening, but also for phenotype discovery and validation, allowing testing of multiple lines, cell types and variables, such as timing and dose-response to perturbagens, including therapeutic agents, pathway and immune modulators, and stress inducers. This is especially important for consideration of the complex biology and genetics underlying many neurological diseases, which will necessitate examination of large numbers of patient hiPSC lines to identify and validate phenotypes, correlate them with genomic variation, and identify new targets for drug discovery.

The data set we have generated regarding modulation of neurites on hiPSC-derived neurons by a comprehensive collection of approved drugs and tool compounds not only serves as an important reference, but also provides evidence that many pathways and targets known to play roles in neurite growth from other studies have similar activities in hiPSC-derived neurons. Further, we demonstrate that these pathways and targets can be identified in an unbiased phenotypic screen of a comprehensive collection of approved drugs and tool compounds for modulators of hiPSC-derived neurons. Our data also suggest that hiPSC-derived neurons provide a useful system to study the mechanisms of action and off-target activities of the approved drugs identified as hits, which could lead to better understanding of their clinical efficacy and toxicity, especially in the context of specific human genetic backgrounds. Finally, the hit set we report constitutes a sublibrary of approved drugs and tool compounds that modulate neurites. This sublibrary will be invaluable for phenotypic analyses and interrogation of hiPSC-based disease models as probes for defining phenotypic differences and cellular vulnerabilities in patient versus control cells, as well as for investigations of the molecular mechanisms underlying human neurite growth in development and maintenance of neuronal networks, and nerve regeneration.

## MATERIALS AND METHODS

### Cell culture

Cryopreserved hiPSC-derived neurons (iCell Neurons; Cellular Dynamics Inc., Madison, WI) were cultured according to the manufacturer's protocols. Neurons were thawed and plated directly onto poly-D-lysine (PDL)-coated 384-well clear-bottom plates (Corning Life Sciences, Tewksbury, MA) at a density of 4000 cells/well, in a volume of 50 µg/well, using a 384-well electronic pipette (Integra Biosciences Corp., Hudson, NH). Neurons were plated in manufacturer-provided medium and then further supplemented with 3.33 µg/ml laminin (Sigma-Aldrich, St Louis, MO), instead of precoating the microplates, to simplify the high-throughput process. Primary screening, hit confirmation and dose response studies were all performed on different batches of iCell Neurons. Assay performance was consistent from batch to batch, based on response to our control compound staurosporine.

### High-content screening assay

Prior to compound addition, 25 µl/well of medium was removed from screening plates (25 µl/well remaining). Compounds solvated in DMSO were transferred to screening plates from 10 mM libraries (50 nl transfer for 5 µM screen, 5 nl transfer for 0.5 µM screen) using an Echo liquid handler (Labcyte Inc., Sunnyvale, CA). Assay plates were backfilled to maintain a constant level of 0.05% DMSO across both concentrations screened. After compound addition, 75 µl/well of fresh medium was added to the screening plate to a final assay volume of 100 µl/well. Assay plates were then incubated for 3 days prior to fixation and immunostaining.

### Compound libraries

Compound libraries screened were selected to represent a diverse set of well-characterized small molecules with a wide range of targets (Table 1). In total, 5215 compounds were screened, representing 4421 unique compounds due to overlap. All compounds were solvated in DMSO. Libraries were screened at two concentrations that we predicted would maximize hit identification and minimize toxicity, based on our historical experience at the Prebys Center using the same bioactive compound libraries on cell-based screens.

### Immunofluorescence

At the endpoint of the screening assay, culture medium was removed and cells were fixed with 4% formaldehyde for 20 min at room temperature. Cells were then washed three times with Dulbecco's phosphate-buffered saline (DPBS) and incubated overnight with primary antibodies in DPBS supplemented with 5% normal donkey serum (Jackson ImmunoResearch Laboratories, Inc., West Grove, PA) and 0.1% Triton X-100 (Sigma-Aldrich). Following primary antibody incubation, plates were washed three times with DPBS and incubated with secondary antibodies and phalloidin where indicated, in DPBS supplemented with 1% normal donkey serum and 0.1% Triton X-100 for 2 h at room temperature. Assay plates were then washed three times with DPBS and nuclei were stained with Hoechst 33342 (5 µg/ml; Thermo Fisher Scientific). All immunofluorescence reagents and dilutions used are described in Table S1.

### High-content imaging and analysis

Assay data were acquired on an Opera confocal microplate imaging system (PerkinElmer, Inc., Waltham, MA) with a 20× air objective. Six fields were acquired per well. All images were analyzed using Columbus Acapella software (PerkinElmer) to identify nuclei and neurite segments. The parameters used in the ‘find nuclei’ block were as follows: method, B; common threshold, 0.40; area >60 µm^2^; split factor, 7.0; individual threshold, 0.40; contrast >0.10. The parameters used for the ‘find neurites’ block were as follows: method, CSIRO Neurite Analysis 2; smoothing width, 3 px; linear window, 9 px; contrast >2; diameter ≥7 px; gap closure distance ≤9 px; gap closure quality, 0; debarb length ≤10 px; body thickening, 5 px; tree length ≤0 px. Data generated in Columbus were exported to CBIS software (ChemInnovation Software, Inc., San Diego, CA) for normalization and hit identification. For analysis of pMLC, neurons were segmented into two regions: cytoplasm (based on TUJ1 staining using ‘find cytoplasm’ building block) and spots positive for phalloidin-stained F-actin (using ‘select cell region’ and ‘find spots’ building blocks). F-actin spots were selected within the extracellular membrane region in order to select regions that correspond to filopodia at points of cell spreading and neurite initiation, as well as growth cones at the tips of neurites. Z-scores were generated to normalize data across plates using the formula 
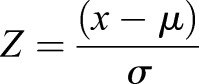
 , where Z is the score for the raw value x, µ and σ represent the mean and standard deviation, respectively, of the vehicle (DMSO) treated control wells. Z-factors were calculated using the formula 
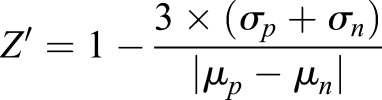
 , where σ represents the means and µ the standard deviations of the positive (p) and negative (n) controls. For calculating Z-factors, staurosporine-treated wells were used as positive controls and vehicle (DMSO)-treated wells as negative controls.

## Supplementary Material

Supplementary information
