# Supplementary material for: High-throughput screen for compounds that modulate neurite growth of human induced pluripotent stem cell-derived neurons
Source: Dis Model Mech. 2018 Feb 1;11(2):dmm031906. doi: 10.1242/dmm.031906 (PMC5894944; doi:10.1242/dmm.031906)
Supplement: Supplementary information [file dmm-11-031906-s1.pdf]

Supplementary Figure and Tables

Figure S1

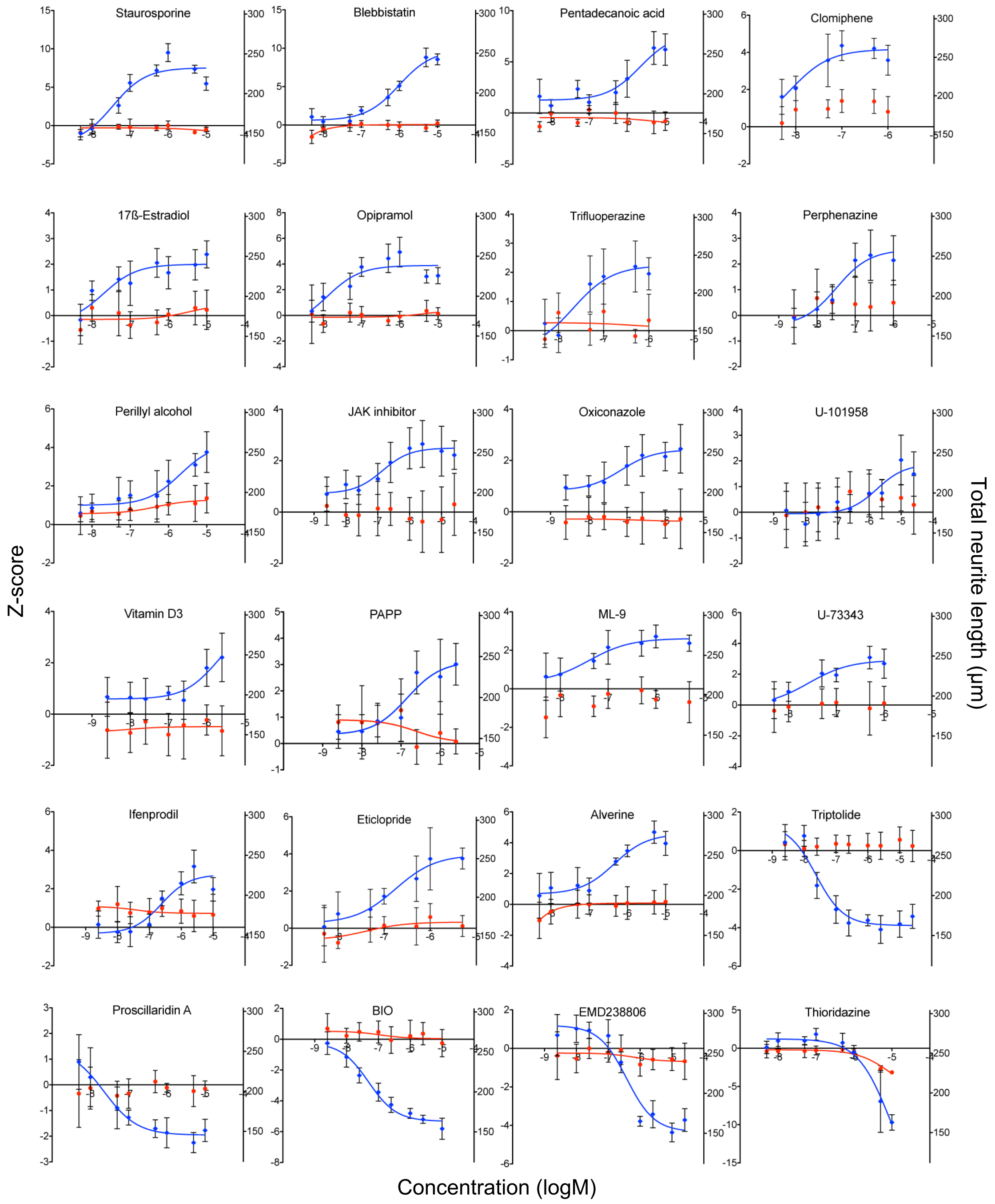

Figure S1. Additional dose-response curves for confirmed hits

Effects on neurite growth (blue: total neurite length) were confirmed at concentrations without negative effects on the number of viable cells (red). Data shown as mean Z-score ±SD, n=6 wells (mean: 244 cells/well). Total neurite length (µm) corresponding to Z-scores is shown on the right of each curve for reference.

Disease Models & Mechanisms • Supplementary information

Table S1. Immunofluorescence reagents

Click here to Download Table S1

Table S2. Primary screen results for cytotoxic compounds

For each compound, the Z-score presented is the average of two duplicates. The Z-score presented is the largest Z-score chosen from the four measured nuclear parameters (Number of nuclei, Nucleus area, Nucleus intensity, & Nucleus roundness).

Click here to Download Table S2

Table S3. Primary screen results for hits promoting neurite outgrowth

For each compound, the Z-score presented is the average of two duplicates at the listed concentration. Compounds that promoted neurite outgrowth at both concentrations tested are listed at each concentration. The Z-score presented is the highest Z-score chosen from the seven measured neurite parameters (Maximum neurite length, Total neurite length, Number of extremities, Number of neurite segments, Number of nodes (type 1), Number of nodes (type 2), & Number of roots).

Click here to Download Table S3

Table S4. Primary screen results for hits inhibiting neurite outgrowth

For each compound, the Z-score presented is the average of two duplicates at the listed concentration. Compounds that inhibited neurite outgrowth without cytotoxic effects at both concentrations tested are listed at each concentration. The Z-score presented is the lowest Z-score chosen from the seven measured neurite parameters (Maximum neurite length, Total neurite length, Number of extremities, Number of neurite segments, Number of nodes (type 1), Number of nodes (type 2), & Number of roots).

Click here to Download Table S4
